# Measurement of Condylar Offset and Posterior Condylar Cartilage Thickness in Normal Knees: An MRI Study From Saudi Arabia

**DOI:** 10.7759/cureus.52244

**Published:** 2024-01-14

**Authors:** Wazzan Aljuhani, Mohammed Alsalman, Hashim Alsalman, Feras O Aljurayyad, Mohammed N Alsubaie, Abdullah Alanazi, Bandar Ahmed

**Affiliations:** 1 Orthopaedics, National Guard Health Affairs, Riyadh, SAU; 2 Radiology, National Guard Health Affairs, Riyadh, SAU; 3 College of Medicine, King Saud Bin Abdulaziz University for Health Sciences, Riyadh, SAU; 4 Department of Surgery, King Abdullah Bin Abdulaziz University Hospital, Riyadh, SAU

**Keywords:** total knee arthroplasty, knee replacement, posterior condylar cartilage, posterior condylar offset, magnetic resonance imaging

## Abstract

Background

The maximum amount of knee flexion after total knee replacement is largely determined by the knee’s posterior condylar offset (PCO). Using magnetic resonance imaging (MRI), this study examined the relationship between PCO and the thickness of the femoral posterior condylar cartilage (PCC) in healthy people.

Methodology

We reviewed the medical records of 300 skeletally mature patients who did not exhibit symptoms of knee arthritis and had undergone MRI for traumatic soft tissue knee injuries that did not affect the femoral PCC.

Results

The study cohort consisted of 300 participants, of whom 68.3% (205) were male, and 31.7% (95) were female aged between 18 and 59 years, with a mean age of 31.13 ± 8.83 years. Most participants were under 30 years of age (45.7%), and the mean body mass index was 27.52 ± 5.64 kg/m^2^. The total medial distance was 28.50 ± 3.11 mm, ranging from 21.20 to 39.80 mm. The medial PCC was 1.71 ± 0.63 mm, ranging from 0.60 to 4.00 mm. The medial bony PCO was 38.40 mm, ranging from 18.80 to 38.40 mm. The total lateral distance was 25.24 ± 3.16 mm, ranging from 13.50 to 34.90 mm. The lateral PCC was 1.48 ± 0.75 mm, ranging from 0.30 to 10.70 mm. Finally, the lateral bony PCO was 23.76 ± 3.19 mm, ranging from 11.99 to 32.8 mm. There was a statistically significant weak positive relationship between the bony lateral PCO and the patients’ age in females only (p = 0.016; r = 0.00-0.39). There was a statistically significant mean difference in the total medial distance, medial PCC, and lateral PCC between the two knees (p < 0.05).

Conclusions

These findings shed light on the factors influencing these parameters, offer insightful information about the associations between particular patient characteristics and knee measurements, and may help guide clinical evaluations and treatment decisions.

## Introduction

The posterior condylar offset (PCO) of the knee is defined as the maximum thickness of the posterior condyle projected posteriorly to the tangent of the posterior cortex of the femoral shaft. This measurement holds significance in total knee arthroplasty (TKA) as it plays a crucial role in determining the maximum achievable knee flexion. The PCO serves as a reliable reference for guiding the rotation of the femoral component, particularly in arthritic knees undergoing TKA [1-3]. Numerous studies have emphasized the importance of restoring the alignment between the femoral shaft and the posterior articular surface of the femur. This alignment is crucial for minimizing flexion instability, optimizing the range of motion, preventing impingement, and enhancing knee kinematics [4-7]. Nevertheless, in cases where there is uneven loss of femoral posterior condylar cartilage (PCC), the accuracy of the posterior condylar axis as a measurement diminishes, particularly in instances such as valgus knees [8].

By focusing on prosthesis design, correct sizing, and precise positioning and rotation of the femoral component, the PCO can be effectively restored during total TKA. Surgeons need to carefully rotate the femoral component and perform necessary ligament releases, as many existing prosthetic knee designs often overlook the size variations between the medial and lateral femoral condyles [9-11]. Research indicates that achieving proper rotation of the femoral component is essential after TKA to minimize complications related to the patella and enhance stability in the knee [12,13]. The femoral component should ideally be rotated parallel to the femur’s transepicondylar axis. Lakstein et al. [14] identified femoral component internal rotation as a major cause of knee dysfunction in patients undergoing revision TKA. Furthermore, femoral component malrotation has been linked to coronal plane instability [15].

Traditional approaches for calculating PCO rely on flawless lateral plain radiographs of the knee. However, these methods do not account for factors such as the thickness of the articular cartilage or potential flaws in the radiographic process. Clarke [16] found a significant variation in the thickness of the articular cartilage in the posterior condyles in his analysis of cartilage removed during primary TKA. The mean cartilage thicknesses on the posterior medial and lateral femoral condyles were found to be 1.7 mm and 2.0 mm, respectively, with a range of 0 to 4 mm. Unlike radiography, we assert that magnetic resonance imaging (MRI) has the capability to visualize the articular cartilage, overcoming imprecision arising from magnification and obliquity. This enables accurate and independent measurements of the true PCO for each femoral condyle. The goal of this study was to determine the normal PCO of the medial and lateral femoral condyles in normal and non-arthritic knees and examine the differences between the medial and lateral PCO.

## Materials and methods

In this retrospective cohort study, we reviewed the medical records of 300 individuals.

Inclusion and exclusion criteria

We included the MRI scans of skeletally mature patients who had MRIs reported to be normal. Individuals with a past medical history of osteochondral abnormalities, deformity, dysplasia, fractures, arthritis, or knee surgery were not eligible.

Data collection and MRI procedure

MRI Technique

Patients underwent routine MRI of the knee with full extension using multiple MRI machines (Phillips Achieva 3 T scanner, Philips Ingenia 3 T scanner (Philips, Amsterdam, The Netherlands), GE Optima 3 T (Boston, MA, USA), and Siemens Magnetom Espree 1.5 T (Munich, Germany)).

Standard sequences included sagittal T2-weighted multi-echo (repetition time (TR)/echo time (TE), 3,200/60), sagittal and coronal proton-spin echo (TR/TE, 3,400/36), and coronal and axial fat-saturated proton-spin echo (TR/TE, 3,500/36) sequences. The slice thickness was 3 mm with a 3.3 mm gap. The field of view was 18 cm and the acquisition matrix size was 320 × 224.

MRI Review and Measurement

**Figure 1:**
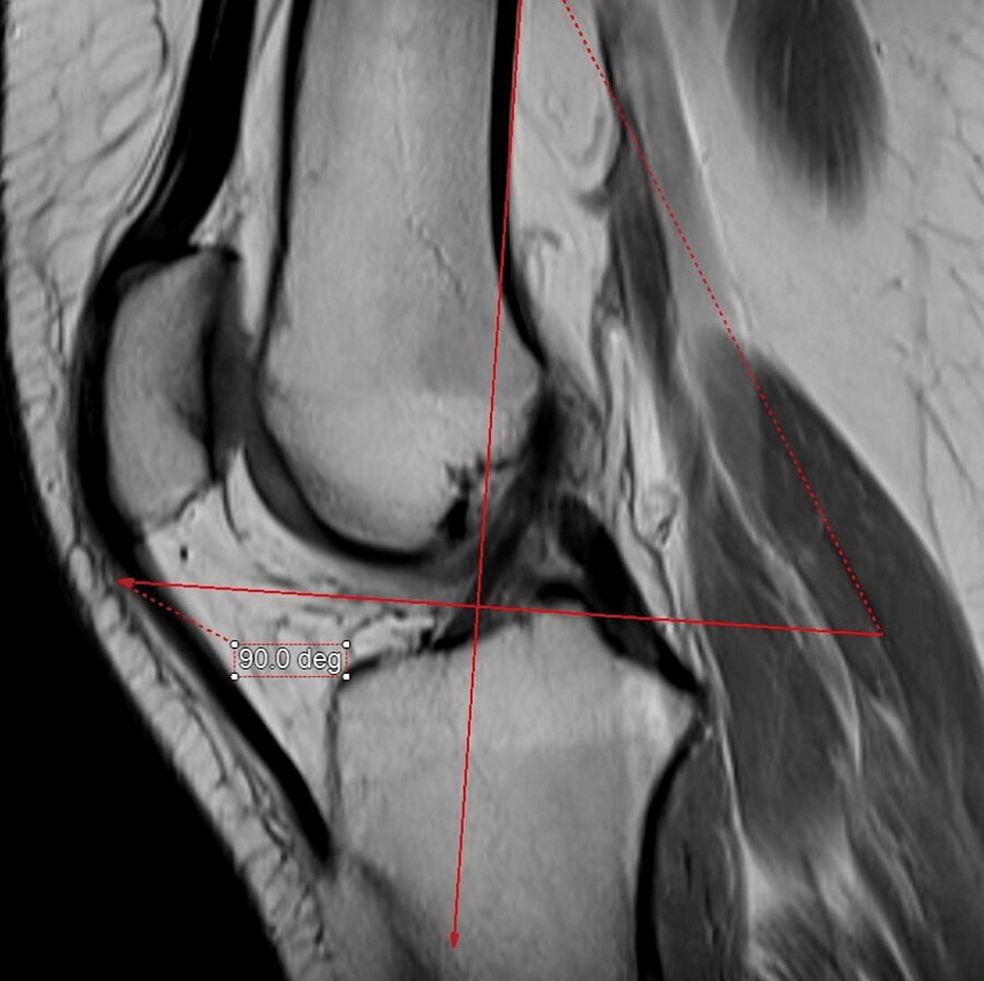
Sagittal proton-density MRI of the knee at the midsagittal plane. Sagittal proton-density MRI of the knee. The femoral posterior cortical axis was identified and drawn in the midsagittal plane.

**Figure 2:**
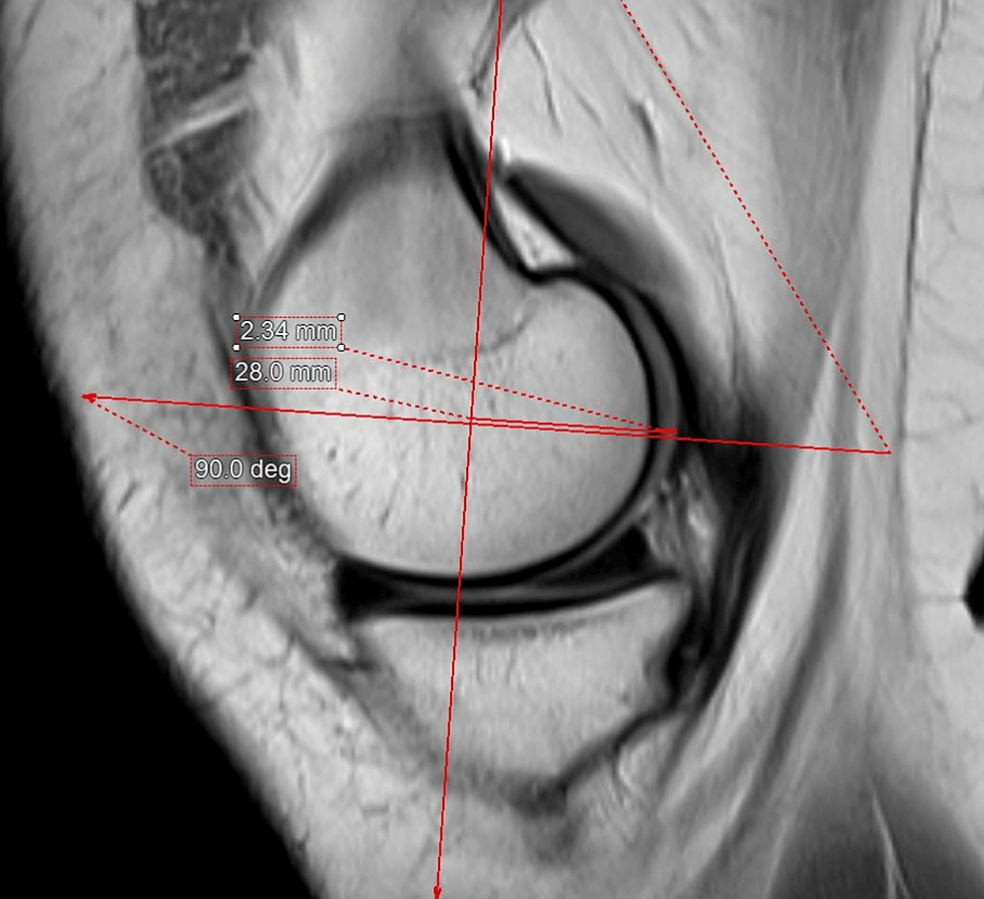
Sagittal proton-density MRI of the knee at the deepest part of the medial femoral condyle. The midsagittal axis is translated medially to measure the posterior condylar offset and posterior condylar cartilage thickness at the deepest part of the posterior condyle perpendicular to the drawn femoral posterior cortical axis.

**Figure 3:**
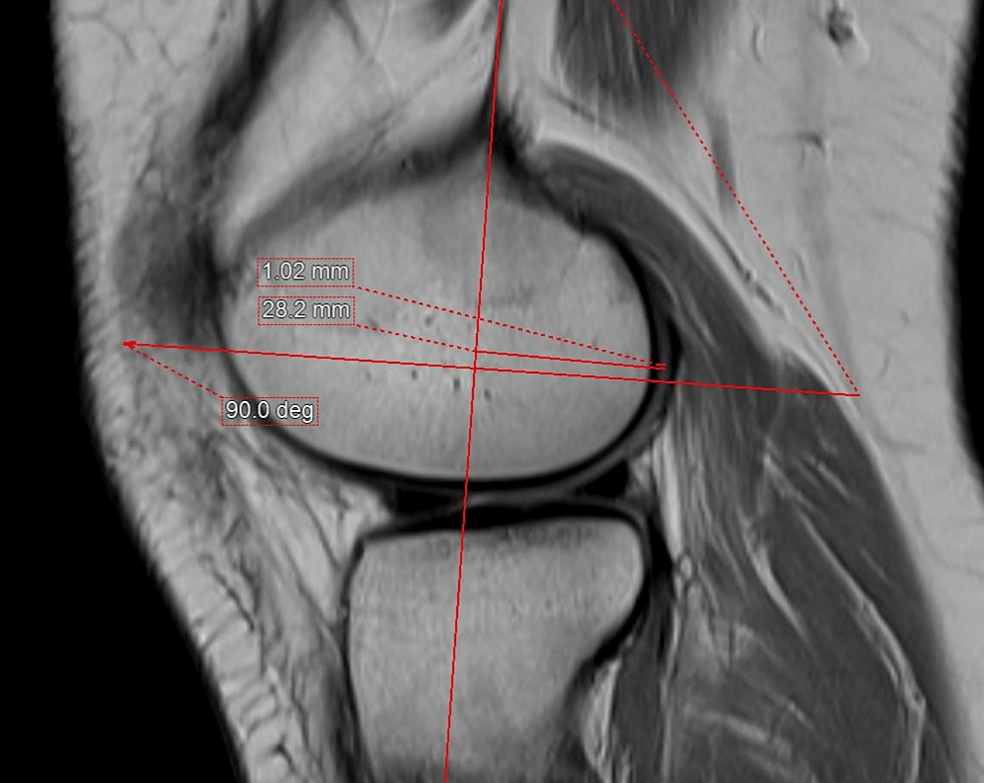
Sagittal proton-density MRI of the knee at the deepest part of the lateral femoral condyle. The midsagittal axis is translated laterally to measure the posterior condylar offset and posterior condylar cartilage thickness at the deepest part of the posterior condyle, perpendicular to the drawn femoral posterior cortical axis.

The medial and lateral PCOs and PCC were measured by three experienced musculoskeletal radiologists. The sagittal proton-density MRI of 300 skeletally mature patients who underwent routine MRI was used for measurement using an integrated third-party volume-rendering software (Intuition 4.6, TeraRecon, Durham, North Carolina, USA) through the Change PACS software (USA). First, the femoral posterior cortical axis was identified in the midsagittal plane (Figure 1). This axis was then translated medially and laterally to the slice chosen to measure the thicknesses of the PCO and PCC (Figures 2, 3). These points were then examined in the axial plane to ensure they were properly centered on their respective condyles. In the sagittal plane, the deepest sections of the medial and lateral posterior condyles were detected. Two measurements perpendicular to the posterior cortical axis were taken in these MRI slices (Figures 2, 3), i.e., the distance from the posterior cortical axis to the articular surface and the PCC thickness at this level. The bone PCO was computed by subtracting the PCC thickness from the entire distance [17]. An orthopedic specialist then examined the femoral PCC thickness and PCO, which were rounded to 0.01 mm [18].

Statistical analysis

The information collected was placed into a secure database and analyzed using SPSS version 26 (IBM Corp., Armonk, NY, USA). Simple descriptive statistics were employed to determine sociodemographic features using counts and percentages for categorical variables and means and standard deviations for continuous variables. The chi-square test was performed to establish the relationship between categorical variables in terms of correlation. Pearson’s correlation coefficient was utilized to correlate the variables represented by means. Student’s t-test was used to compare the means of the PCC and PCO readings. These tests were performed under the assumption of a normal distribution. Finally, p-values <0.05 were utilized to rule out the null hypothesis.

Ethical approval

This study was approved by the Research Ethics Committee of the King Abdullah International Research Center (IRB NRC23R/150/03) on 03/08/2023. Due to the nature of the study, informed consent was waived.

## Results

The study sample comprised 300 participants, with 68.3% males and 31.7% females aged from <30 to >40 years, with a mean age of 31.13 ± 8.83 years. The mean height of the participants was 166.56 ± 9.23 cm, while the mean weight was 76.48 ± 17.39 kg.

A nearly equal representation was observed for the right (52.0%) and left (48.0%) knees. Table 1 provides insights into the distribution of participants among different MRI machine types, including Achieva, GE, GE Discovery, GE Genesis, GE Optima, Ingenia, Panorama, Siemens, Siemens Espree, and Siemens Vida. Siemens Espree had the highest representation, accounting for 32.7% of the participants. The mean total distance for the medial aspect of the knee was 28.50 mm, ranging from 21.20 to 39.80 mm. The mean PCC for the medial aspect was 1.71 mm, ranging from 0.60 to 4.00 mm. The mean bony PCO for the medial aspect was 26.78 mm, ranging from 18.80 to 38.40 mm. The mean PCC for the lateral aspect was 1.48 mm, ranging from 0.30 to 10.70 mm. Finally, the mean bony PCO for the lateral aspect was 23.76 mm, ranging from 11.99 to 32.80 mm (Table 1).

**Table 1:** Demographics and study factors. All values are presented as numbers and percentages for categorical variables and mean and standard deviations for numerical variables. BMI = body mass index; PCO = posterior condylar offset; PCC = posterior condylar cartilage; SD = standard deviation

Characteristics (n 300)	n (%)
	Mean ± SD
Gender	
Male	205 (68.3)
Female	95 (31.7)
Age (years)	31.13 ± 8.831
<30	137 (45.7)
30–34	71 (23.7)
35–40	38 (12.7)
>40	54 (18.0)
Height (cm)	166.56 ± 9.23 cm
Weight (kg)	76.48 ± 17.39 kg
BMI	27.52 ± 5.64 kg/m^2^
Underweight	9 (3.0)
Normal weight	96 (32.0)
Overweight	111 (37.0)
Obese	84 (28.0)
Knee side	
Right	156 (52.0)
Left	144 (48.0)
MRI machine	
Achieva	102 (34.0)
GE	2 (0.7)
GE Discovery	10 (3.3)
GE Genesis	2 (0.7)
GE Optima	14 (4.7)
Ingenia	66 (22.0)
Panorama	4 (1.3)
Siemens	1 (0.3)
Siemens Espree	98 (32.7)
Siemens Vida	1 (0.3)
Medial total distance (mm), (mean ± SD)	28.50 ± 3.11 mm
Range (mm)	21.20–39.80 mm
Medial PCC (mm), (mean ± SD)	1.71 ± 0.63 mm
Range (mm)	0.60–4.00 mm
Medial bony PCO (mm), (mean ± SD)	26.78 ± 3.12 mm
Range (mm)	18.80–38.40 mm
Lateral total distance (mm), (mean ± SD)	25.24 ± 3.16 mm
Range (mm)	13.50–34.90 mm
Lateral PCC (mm), (mean ± SD)	3.48 ± 0.75 mm
Range (mm)	0.30–10.70 mm
Lateral bony PCO (mm), (mean ± SD)	23.76 ± 3.19 mm
Range (mm)	11.99–32.80 mm

Table 2 presents the relationship and the correlation between the medial bony PCO and lateral bony PCO with the weight depending on the patients’ sex. There were no statistically significant relations between any of the factors and the patients’ weight (p > 0.05).

**Table 2:** The relationship between the medial bony PCO and lateral bony PCO with weight depending on the patients’ sex using simple linear regression analysis. Not statistically significant at the 0.05 level of significance. PCO = posterior condylar offset

Weight		Pearson correlation	Regression	
			F-score	P-value
Male	Medial bony PCO	-0.38	0.295	0.587
	Lateral bony PCO	0.07	1.013	0.315
Female	Medial bony PCO	-0.03	0.070	0.791
	Lateral bony PCO	0.10	0.945	0.334

Table 3 describes the relationship and the correlation between the medial bony PCO and lateral bony PCO with height, depending on the patients’ sex. There was a statistically significant weak positive relationship between the medial bony PCO and lateral bony PCO with the patients’ height in males only (p < 0.05; r = 0.00-0.39).

**Table 3:** The relationship between the medial bony PCO and lateral bony PCO with height depending on the patients’ gender using simple linear regression analysis. *: Statistically significant at the 0.05 level of significance. PCO = posterior condylar offset

Height		Pearson correlation	Regression	
			F-score	P-value
Male	Medial bony PCO	0.15	4.863	0.029*
	Lateral bony PCO	0.25	13.569	<0.001*
Female	Medial bony PCO	0.05	0.192	0.662
	Lateral bony PCO	0.16	2.433	0.122

Table 4 describes the relationship and correlation between the medial bony PCO and lateral bony PCO with age, depending on the patients’ sex. There was a statistically significant weak positive relationship between the lateral bony PCO and the patients’ age in females alone (p = 0.016; r = 0.00-0.39).

**Table 4:** The relationship between the medial bony PCO and lateral bony PCO with age depending on the patients’ gender using simple linear regression analysis. *: Statistically significant at the 0.05 level of significance. PCO = posterior condylar offset

Age		Pearson correlation	Regression	
			F-score	P-value
Male	Medial bony PCO	0.10	1.915	0.168
	Lateral bony PCO	0.12	2.917	0.089
Female	Medial bony PCO	-0.01	0.005	0.942
	Lateral bony PCO	0.25	6.07	0.016*

Table 5 presents the mean differences between the medial PCC, medial bony PCO, total medial distance, lateral PCC, lateral bony PCO, and total lateral distance within the patients’ gender. There were statistically significant mean differences with all factors and the patients’ gender (p < 0.05).

**Table 5:** The relationship between the medial PCC, medial bony PCO, medial total distance, lateral PCC, lateral bony PCO, and total lateral distance with gender using the independent-samples t-test. All values are means and SDs. *: Statistically significant differences at the 0.05 level of significance. PCO = posterior condylar offset; PCC = posterior condylar cartilage; SD = standard deviation

Factors	Mean	SD	t	P-value
Medial PCC (mm)				
Male	1.79	0.66	3.008	0.003*
Female	1.55	0.54		
Medial bony PCO (mm)				
Male	27.15	3.06	3.010	0.003*
Female	25.99	3.14		
Medial total distance (mm)				
Male	28.9	3.01	3.661	<0.001*
Female	27.6	3.13		
Lateral PCC (mm)				
Male	1.57	0.84	3.323	0.001*
Female	1.27	0.46		
Lateral bony PCO (mm)				
Male	24.48	3.09	6.046	<0.001*
Female	22.22	2.85		
Lateral total distance (mm)				
Male	26.05	2.98	7.063	<0.001*
Female	23.48	2.82		

Table 6 shows the association between the knee sides and the patients’ demographic factors. There were no statistically significant associations among any of the factors (p > 0.05).

**Table 6:** The association between knee sides and the patients’ demographic factors using the chi-square test. No association was found at the 0.05 level of significance. BMI = body mass index

Demographics	Knee side				P-value
	Right		Left		
	n	(%)	n	(%)	
Gender					
Male	107	68.6	98	68.1	0.921
Female	49	31.4	46	31.9	
Age (years)					
<30	67	42.9	70	48.6	0.747
30–34	40	25.6	31	21.5	
35–40	21	13.5	17	11.8	
>40 years	28	17.9	26	18.1	
BMI					
Central region	4	2.6	5	3.5	0.858
Eastern region	49	31.4	47	32.6	
Western region	61	39.1	50	34.7	
Northern region	42	26.9	42	29.2	

The mean differences between the right and left knees for all research parameters are shown in Table 7. Between the two knee sides, there was a statistically significant mean difference in the total medial distance, medial PCC, and lateral PCC (p < 0.05).

**Table 7:** The relationship between the study factors and the knee side using the independent-samples t-test. All values are means and SDs. *Statistically significant differences at 0.05 level of significance. PCO = posterior condylar offset; PCC = posterior condylar cartilage; SD = standard deviation

Factors	Mean	SD	t	P-value
Medial total distance (mm)				
Right	28.84	3.41	1.98	0.049*
Left	28.13	2.71		
Medial PCC (mm)				
Right	1.85	0.61	3.87	<0.001*
Left	1.57	0.63		
Medial bony PCO (mm)				
Right	26.99	3.47	1.20	0.233
Left	26.56	2.69		
Lateral total distance (mm)				
Right	25.31	3.26	0.42	0.676
Left	25.16	3.06		
Lateral PCC (mm)				
Right	1.58	0.91	2.54	0.012*
Left	1.36	0.51		
Lateral bony PCO (mm)				
Right	23.73	3.33	-0.18	0.858
Left	23.80	3.05		

## Discussion

It is critical in TKA to assess PCC thickness and symmetry between the medial and lateral condyles. In TKA, the PCO influences the extent of potential knee flexion. A 1-mm drop in PCO following TKA reduced knee flexion by 6.1°. Massin and Gournay [7] discovered that a 3-mm decrease in the PCO after TKA could reduce knee flexion by 10° before tibiofemoral impingement occurs [15]. The total PCO is composed of the PCC thickness and bony condyles [19,20]. Kinematic TKA necessitates knowledge of the anatomy of the typical condylar and articular cartilages. The posterior femoral joint line was repaired by precisely resecting the bone and cartilage to match the femoral component thicknesses. As a result, PCC should be taken into account when selecting resection depth [21,22]. Understanding the anatomy of the typical condylar and articular cartilages is required for kinematic TKA. By precisely resecting the bone and cartilage to match the thicknesses of the femoral components, the posterior femoral joint line was restored. As a result, PCC should be taken into account while establishing the resection depth. We found a statistically significant weak correlation between the medial bony PCO and lateral bony PCO and the patients’ height. Additionally, there was a statistically significant weak correlation between the lateral bony PCO and the patients’ age in females alone. Within the patients’ sex, there were statistically significant mean differences in the medial PCC, medial bony PCO, total medial distance, lateral BCC, lateral bony PCO, and total lateral distance. There was no statistically significant association between patient demographics and the knee side. Statistically significant mean differences were observed between the two sides of the knees (total medial distance, medial PCC, and lateral PCC).

Indeed, the majority of anatomic studies have been conducted in arthritic individuals with cartilage and bony erosion, and measurements are typically made using radiography and computed tomography, which may underestimate the normal PCO [8-10]. Voleti et al. [17] examined 32 patients without a history of knee pathology who underwent both plain radiography and MRI scans of the same knee. PCO was assessed on lateral radiographs and compared with MRI measurements using an innovative three-dimensional protocol. The mean medial PCO was 29 ± 3 mm, and the mean lateral PCO was 26 ± 3 mm; both values exceeded the mean radiographic PCO of 25 ± 2 mm. Notably, the medial PCO, as measured by MRI, was significantly greater than the lateral PCO.

The PCO of normal and non-arthritic knees varied greatly in size. The range of measurements employed in this study varied greatly depending on the different features. The range for total medial distance medial was 21.20-39.80 mm. The medial PCC ranged from 0.60 to 4.00 mm, indicating moderate diversity. The range for medial bony PCO was 18.80-38.40 mm. Shifting to the lateral aspect, the total lateral distance spanned from 13.50 to 34.90 mm. The lateral PCC had a range of 0.30-10.70 mm. Finally, for the lateral bony PCO, the range was 11.99-32.80 mm. These ranges provide insights into the spread of data points within each specific aspect and offer a clear picture of the diversity and distribution of these measurements. Wernecke et al. [23] used MRI to determine the associations between femoral PCC thickness and PCO in 530 healthy subjects. The authors reviewed the records of 287 male and 243 female patients who underwent MRIs for traumatic soft tissue knee injuries that did not include the femoral PCC and did not have symptomatic knee arthritis. The lateral and medial PCC thicknesses (2.04 vs. 1.99 mm) were comparable with no statistically significant difference. Males had a significantly thicker PCC on the medial and lateral sides than females (2.05 vs. 1.92 mm and 2.16 vs. 1.86 mm, respectively). The PCC thickness did not correlate with age. They also found that bony PCO was greater on the medial side than on the lateral side (25.8 vs. 22.6 mm). According to Schub et al. [18], the average mean thickness of the knee cartilage at all measured sites was approximately 1.27 mm, with the thinnest areas suitable for matching found at approximately 1.95 mm of the posterior pole of the medial femoral condyle and 1.85 mm of the distal-most anterior-lateral femoral condyle.

Strengths and limitations

It is important to acknowledge certain limitations of this study. One notable restriction is that all imaging studies were conducted at a single medical site. However, the study had several strengths, including a large sample size, which helped mitigate the risk of sampling bias. Additionally, to our knowledge, this is the first study in Saudi Arabia to explore this topic.

## Conclusions

Remarkably, we discovered a statistically significant weak correlation between the medial and lateral bony PCO and the height of patients. However, this correlation was observed specifically in males. Likewise, a statistically significant weak correlation between lateral bony PCO and the age of patients was identified exclusively in females. Additionally, we detected significant sex-based variations in various measurements, including the medial BCC, medial bony PCO, total medial distance, lateral BCC, lateral bony PCO, and total lateral distances. However, we did not observe any significant associations between patient demographics and the knee side. Furthermore, our research indicated statistically significant variations in total medial distance, medial PCC, and lateral PCC between the two sides of the knees. These findings provide valuable insights into the relationships between specific patient characteristics and knee measurements, shed light on the factors influencing these parameters, and can potentially inform clinical assessments and treatments.
